# Transcriptional Intermediary Factor 1γ–Induced Irisin in Skeletal Muscle Attenuates Renal Fibrosis in Diabetic Nephropathy

**DOI:** 10.1002/jcsm.13810

**Published:** 2025-04-15

**Authors:** Jin Hyun Kim, Seunghye Lee, Hani Jang, Sehyun Jung, Myeong Hee Jung, Jeong Won Yun, Haejin Jeon, Hyun‐Jung Kim, Se‐Ho Chang, Eun Ju Lee, Hyo‐Soo Kim

**Affiliations:** ^1^ Biomedical Research Institute Gyeongsang National University Hospital Jinju Republic of Korea; ^2^ Institute of Medical Science Gyeongsang National University Jinju Republic of Korea; ^3^ Division of Nephrology, Department of Internal Medicine Gyeongsang National University College of Medicine and Gyeongsang National University Hospital Jinju Republic of Korea; ^4^ Biomedical Research Institute Seoul National University Hospital Seoul Republic of Korea; ^5^ Molecular Medicine & Biopharmaceutical Sciences Seoul National University Seoul Republic of Korea; ^6^ Department of Internal Medicine Seoul National University College of Medicine Seoul Republic of Korea

**Keywords:** chronic kidney disease, diabetic nephropathy, fibrosis, irisin, muscle‐kidney crosstalk, transcriptional intermediary factor 1γ

## Abstract

**Background:**

Transcriptional intermediary factor 1γ (TIF1γ) is a negative regulator of TGF‐β1 signalling and has been associated with patient survival in renal cell carcinoma. However, its role in diabetes mellitus (DM), particularly in diabetic nephropathy (DN), remains unclear. DN is the leading cause of chronic kidney disease (CKD). We investigated the potential role of TIF1γ in mitigating multiple DM‐related complications.

**Methods:**

Mice were divided into four groups: *db*/m+, *db/db* and *db/db* mice treated with cytomegalovirus‐ or TGF‐TIF1γ plasmids (40 μg/mouse; intraperitoneally weekly for 16 weeks). Renal injury, fibrosis, function and gene expression related to fibrosis and epithelial–mesenchymal transition (EMT) in the kidneys were assessed. Muscle atrophy, regeneration markers, myokine levels and exercise capacity were evaluated. C2C12 cells were exposed to palmitate with or without TIF1γ transfection, and irisin expression and secretion were measured. Muscle‐kidney crosstalk was analysed using conditioned media (CM) from TIF1γ‐transfected C2C12 cells in palmitate‐treated human kidney (HK)‐2 cells. Additionally, HK‐2 cells were incubated in CM from fibronectin type III domain‐containing protein (FNDC)5‐knockdown C2C12 cells to confirm irisin‐mediated kidney crosstalk by TIF1γ.

**Results:**

TIF1γ treatment in *db/db* mice resulted in a significant attenuation of renal tubulointerstitial fibrosis (1.5‐fold decrease), glomerular injury (1.8‐fold improvement), tubular injury (1.6‐fold improvement), renal dysfunction (1.7‐fold improvement) and a reduction in EMT‐related factors (1.8‐fold decrease) (*p* < 0.05). The levels of administered TIF1γ plasmids were higher in skeletal muscle than in renal tissues. TIF1γ expression was significantly elevated in the skeletal muscle of *db/db* mice treated with TIF1γ plasmids (6.5‐fold) (*p* < 0.05). Mice receiving both plasmids exhibited a 1.8‐fold reduction in pathological muscle morphology and atrophy‐related gene expression, a 3.0‐fold increase in regeneration‐related gene expression and a 1.6‐fold improvement in muscle function (*p* < 0.05). Irisin expression increased by 2.1‐fold in skeletal muscle and serum (*p* < 0.05). In TIF1γ‐transfected C2C12 cells, irisin secretion was elevated by 1.5‐fold (*p* < 0.05). CM from TIF1γ‐transfected C2C12 cells attenuated EMT in palmitate‐treated HK‐2 cells, compared with medium from nontransfected C2C12 cells (1.9‐fold improvement [*p* < 0.05]). Conversely, FNDC5 knockdown in C2C12 cells accelerated EMT in palmitate‐treated HK‐2 cells, as evidenced by decreased bone morphogenetic protein‐7 (1.6‐fold) and increased EMT‐related factors (2.1‐fold) (*p* < 0.05), compared with palmitate alone and small interfering RNA control.

**Conclusions:**

Our findings emphasize the potential of TIF1γ as a multitargeted therapeutic agent for DN, mitigating both renal and muscular complications through direct fibrosis inhibition and indirect myokine‐mediated inter‐organ crosstalk.

## 1 Introduction

Chronic kidney disease (CKD) is a growing global health concern, affecting over 800 million individuals worldwide [1]. Despite significant efforts to mitigate its impact, improvements in CKD incidence and mortality rates have been modest in recent decades. No universally effective therapeutic strategies have been established to prevent kidney damage [2]. Therefore, the identification and validation of novel therapeutic interventions to enhance the prognosis of CKD patients are of paramount importance.

Diabetic nephropathy (DN), a major microvascular complication of diabetes mellitus (DM), is the leading cause of CKD [3]. Notably, hyperglycaemia is a significant independent risk factor for rapid CKD progression [4, 5]. Long‐term metabolic disturbances in DM, including hyperglycaemia and hyperaminoacidaemia, contribute to intraglomerular hypertension by altering renal hemodynamics, inducing inflammation and promoting fibrosis, ultimately leading to end‐stage renal disease [3, 6]. A pathological hallmark of CKD is transforming growth factor (TGF)‐β‐mediated interstitial fibrosis, a critical factor in its onset and progression [7]. Epithelial–mesenchymal transition (EMT) plays a pivotal role in renal fibrosis and CKD progression. DN is characterized by EMT of tubular epithelial cells and podocytes, leading to tubulointerstitial fibrosis and glomerulosclerosis, respectively. The TGF‐β1/Smad and Wnt/β‐catenin pathways are the primary mechanisms driving EMT in tubular epithelial cells and podocytes [8].

Transcriptional intermediary factor 1γ (TIF1γ), also known as TRIM33, RFG7, PTC7 or ectodermin, is a member of the E3 ubiquitin‐ligase family, characterized by a RING‐box‐coiled‐coil region. It functions as a negative regulator of TGF‐β1 signalling, one of the most potent profibrotic pathways, by promoting SMAD4 ubiquitination and facilitating the turnover of TGF‐β receptors [9, 10]. Structurally, TIF1γ proteins are highly conserved and typically contain a RING domain, which is implicated in ubiquitination. TIF1γ proteins play a critical role in various cellular processes, including viral immunity, inflammatory responses, autophagy and tumour progression [11]. Although the role of TIF1γ in cancer and myositis has been extensively investigated, studies evaluating its role in kidney disease remain limited and focused on renal cell carcinoma [12].

Several studies investigating muscle–organ crosstalk have demonstrated interconnections between skeletal muscles, brain cells and adipose tissues [13, 14]. For instance, skeletal muscle activity influences metabolic changes in brain cells and adipose tissues. Regular physical exercise mitigates the risk of progressive CKD [15], suggesting a functional interaction between skeletal muscles and the kidneys. Notably, patients with DN frequently exhibit comorbid metabolic derangements affecting kidneys and multiple organ systems, including skeletal muscles. Therefore, a multiorgan therapeutic strategy is needed to address DN and its systemic manifestations. We investigated the potential of TIF1γ as a novel therapeutic strategy to ameliorate DM‐related complications in the kidneys and muscles of *db/db* mice, a model of type 2 DM (T2DM)–induced CKD.

## 2 Materials and Methods

### 2.1 Animals, Surgery and Tissue Preparation

All animal experimental protocols were approved by the Gyeongsang National University Institutional Animal Care and Gyeongsang National University Institutional Ethics Committee (GNU‐210202‐M0011–01). The methods were carried out in accordance with the regulations and guidelines established by this committee.

Male *db*/m+ and *db/db* mice (5 weeks of age) were maintained in a temperature (23 ± 1°C)‐ and humidity (55 ± 5%)‐controlled facility with a 12 h/12 h light/dark cycle. Standard mice chow and water were provided *ad libitum*. Mice were assigned to four groups: *db*/m+ mice (non‐diabetic, normal mice; *n* = 5), *db/db* mice (untreated mice, *n* = 7) and *db/db* mice treated with cytomegalovirus (CMV)‐TIF1γ (*n* = 10) or TGF‐TIF1γ‐plasmids. TIF1γ plasmids (40 μg/mouse) were administered intraperitoneally once weekly for 16 weeks. The CMV/TGF‐β primer‐driven TIF1γ‐encoding plasmids were provided by Professor Hyo‐Soo Kim and Eun Ju Lee at Seoul National University Hospital. Mice were anaesthetised with an intraperitoneal injection of Avertin (2,2,2‐tribromoethanol; Sigma‐Aldrich, St. Louis, MO, USA), and blood, kidney and skeletal muscle tissues were harvested.

### 2.2 Histopathology

Tissues were routinely fixed in 4% phosphate‐buffered paraformaldehyde and paraffin‐embedded, and 5‐μm thick sections were cut. Paraffin wax was removed with xylene, and sections were rehydrated with ethanol. Sections were stained with Periodic Acid‐Schiff (PAS) for histopathological analysis and Masson's trichrome (MT) staining to assess tissue fibrotic changes. Semiquantitative scoring was performed for haematoxylin and eosin (H&E) staining to examine the degree of injury, which was assigned points (0–3) based on the extent of interstitial fibrosis, tubular atrophy (defined as luminal dilation and flattened tubular epithelial cells) and interstitial inflammatory cell infiltration. Tissue injury, including interstitial fibrosis, tubular atrophy and interstitial inflammatory cell infiltration, was scored by grading the percentage of affected under a high‐powered field (× 400) as follows: score of 0, 0%; score of 1, < 30%; score of 2, 31%–60%; and score of 3, 61%–100%. All scores were summed and presented as average values on the graph. Signals were analysed using NIS‐Elements BR 3.2 (Nikon, Tokyo, Japan).

### 2.3 Immunoblotting

Tissues were homogenized in RIPA buffer (Thermo Scientific, Waltham, MA, USA). Proteins (50 μg) were loaded and electroblotted. The blots were probed with primary antibodies against anti‐TGF‐β1 (Santa Cruz Biotechnology, Santa Cruz, CA, USA), α‐smooth muscle actin (α‐SMA) (Sigma‐Aldrich), fibronectin type III domain‐containing protein 5 (FNDC5) (Abcam, Cambridge, UK), TIF1γ (Abcam), peroxisome proliferator‐activated receptor gamma coactivator 1‐alpha (PGC‐1α) (Abcam), αB‐crystallin (Enzo Biochem Inc. Farmingdale, NY, USA), phosphorylated Smad 2/3 (pSmad2/3) (Santa Cruz Biotechnology), bone morphogenetic protein 7 (BMP7) (Santa Cruz Biotechnology), brain‐derived neurotrophic factor (BDNF) (Abcam) and fibroblast growth factor‐21 (FGF‐21) (Boster Bio, Pleasanton, CA, USA) at 4°C overnight. The primary antibody was visualized by a secondary antibody and an ECL kit (Amersham Pharmacia Biotech, Piscataway, NJ, USA). The β‐actin antibody (Sigma‐Aldrich) served as the loading control. Densitometric analysis was performed for quantitative analysis of all data.

### 2.4 Immunohistochemistry

For immunohistochemistry studies, an avidin‐biotinylated‐HRP (ABC) (Vector Laboratories, Burlingame, CA, USA) kit was used along with 5 μm‐thick tissue sections. After incubation with 1% normal serum, sections were exposed to primary antibodies against anti‐α‐SMA (Sigma‐Aldrich), nephrin (Abcam) and podocin (Abcam) at 4°C. They were washed with PBS (pH 7.4), incubated with a secondary antibody for 90 min and then incubated with ABC for 60 min at room temperature. Following PBS rising, reactions were developed using 0.027% 3,3‐diaminobenzidine tetrahydrochloride (Sigma‐Aldrich) with 0.003% H_2_O_2_. Sections were counterstained with haematoxylin to visualize cell nuclei and subsequently visualized via light microscopy. Digital images were captured and analysed using NIS‐Elements BR 3.2. Semiquantitative analysis was performed by counting the number and density of positive cells per field in the tissue at × 400 magnification.

### 2.5 Semiquantitative Real‐Time Polymerase Chain Reaction (qPCR)

Tissue samples were obtained for quantitative real‐time PCR. Total RNAs were isolated from kidney and muscle tissues using TRIzol (Invitrogen, Carlsbad, CA, USA). Purified RNAs were reverse transcribed using an iScript cDNA synthesis kit (Bio‐Rad Laboratories, Hercules, CA, USA). Quantitative cDNA amplification was performed using a ViiA7 Real‐Time System (Applied Biosystems Inc. Foster City, CA, USA), a Power SYBR Green PCR Master Mix (Applied Biosystems) and gene‐specific primers. *GAPDH* was used as an internal control to normalize the quantity of RNA. Relative gene expression level in each sample was quantified using the 2^−ΔΔCt^ method. The list of the primer sequences used is presented in Table 1.

**TABLE 1:** List of primer sequences used in this study.

Genes	Forward primer (5′‐3′)	Reverse primer (5′‐3′)
*TGF‐β1*	TGCGCTTGCAGAGATTAAAA	CGTCAAAAGAGACAGCCACTCA
*Twist*	CTCGGACAAGCTGAGCAAG	CAGCTTGCCATCTTGGAGTC
*Atrogen1*	ATG CAC ACT GGT GCA GAG AG	TGT AAG CAC ACA GGC AGG TC
*MuRF1*	ACC TGC TGG TGG AAA ACA TC	AGG AGC AAG TAG GCA CCT CA
*MyoD1*	CAA GCG CAA GAC CAC CAA CG	ATA TAG CGG ATG GCG TTG C
*Mfy5*	CCT CAT GTG GGC CTG CAA A	CAT TCC TGA GGA TCT CCA CC
*FGF‐21*	CTGGGGGTCTACCAAGCATA	CACCCAGGATTTGAATGACC
*BDNF*	GCCCAACGAAGAAAACCATA	GCTGTGACCCACTCGCTAAT
*FNDC5*	GCTAGGCTGCGTCTGCTTC	AGCCAATGACCACTTCATCC
*GAPDH*	ACTCCACTCACGGCAAATTC	TCTCCATGGTGGTGAAGACA

### 2.6 Hanging Grid Test

Inverted hanging time was measured using a modified version of the test by Shang et al. [16]. Briefly, a 45 × 45 cm grid (bar thickness, 2 mm; mesh, 18 mm) was placed on a 55‐cm high frame, and a 5‐cm thick cushion was placed under the grid. The distance between the grid and the cushion was 50 cm. Each mouse was placed at the centre of the grid, and the grid was turned upside down with the mouse headfirst declining. The hanging time was recorded as the time taken for the mice to fall. Each mouse was tested three times with a > 30‐min interval between tests, and the hanging time was recorded.

### 2.7 Forelimb Grip Strength Test

Forelimb grip strength was measured using a grip strength meter (BIO‐GS3, BIOSEB, Vitrolles, France). Taking advantage of a mouse's instinct to grab anything available while suspended, the mouse was trained to grasp a horizontal bar attached to the dynamometer. Subsequently, the mouse was gently pulled backward along the horizontal plane, parallel to the dynamometer, prompting it to resist the pull. As the force applied to the mice gradually increased, the mouse reached a point where it could no longer resist and released the bar. The force applied to the mouse was equal to the force applied to the horizontal bar by the mouse. The forelimb grip strength of each mouse was tested three times, and the measured values were recorded and averaged. Six consecutive measurements were performed per day at 1‐min intervals, and the investigators were blinded to the group allocation of the mice. Grip strength was normalized to body weight.

### 2.8 Irisin Measurement

Serum irisin levels were measured in mice using a commercial enzyme‐linked immunosorbent assay (ELISA) kit (EK‐067‐29, Phoenix Pharmaceuticals, Burlingame, CA, USA) according to the manufacturer's protocol.

### 2.9 Cell Culture and Treatment

The C2C12 mouse skeletal muscle myoblasts were purchased from the American Type Culture (ATCC, USA). To induce differentiation into myotubes, C2C12 myoblasts were cultured in DMEM supplemented with 2% horse serum. Myotube morphology was observed after 3–4 days, and the cells were used for subsequent in vitro experiments. C2C12 cells were transfected with 2 μg of CMV/TGF‐TIF1γ plasmids using lipofectamine.

Palmitate (PA), a saturated‐free fatty acid, was used to establish an in vitro obesity and lipotoxicity model. Cells were treated with PA at various doses, diluted with a differentiation medium containing 2% fat‐free bovine serum albumin. Cell lysates and culture medium were collected for protein expression and myokine secretion analysis.

To investigate the paracrine effect of C2C12 cells expressing TIF1γ on palmitate‐treated HK‐2, a human proximal tubular cell line, cells in vitro, HK‐2 cells were treated with 100 μM palmitate under various conditions. These conditions were as follows: an α‐MEM medium supplemented with 5% FBS and a conditioned medium from non‐transfected C2C12 cells and those transfected with TIF1γ plasmids for 8, 24 and 48 h. After 24 and 48 h, the expression levels of TIF1γ, the epithelial marker (BMP) and PGC‐1α in HK‐2 cells were investigated using immunoblot analysis.

The small interfering RNA (siRNA) that targets FNDC5, BDNF, FGF‐21 and negative control were obtained from Bioneer (Daejeon, South Korea) to block the FNDC5, BDNF and FGF‐21 expression. Transient transfection was performed using 25 nmol of each siRNA and 6 μL of Lipofectamine RNAiMax (Invitrogen) in a serum‐free medium according to the instructions of the manufacturer. The culture medium and the cells were harvested for 24, 48, 72 h.

### 2.10 Statistical Analysis

Statistical analyses were performed using the GraphPad Prism software (version 9.0; GraphPad Software Inc. La Jolla, CA, USA) and determined using a one‐way analysis of variance followed by Tukey's multiple comparison test. All statistical tests used *p* < 0.05 to indicate significance.

## 3 Results

### 3.1 Effect of TIF1γ on Body Weight and Glucose Homeostasis

To achieve in vivo overexpression of TIF1γ, *db/db* mice were intraperitoneally injected with CMV‐ or TGF‐TIF1γ plasmids (40 μg per mouse), whereas *db/*m+ mice received saline injections (Figure S1A). During the 16‐week TIF1γ plasmid treatment, both untreated and TIF1γ‐treated *db/db* mice exhibited significantly higher body weights than *db/*m+ mice (Figure S1B). Although TIF1γ‐treated *db/db* mice had lower body weights than untreated *db/db* mice, the difference was not statistically significant (Figure S1B). Fasting glucose levels increased with age in all *db/db* mice (Figure S1C). Both untreated and TIF1γ‐treated *db/db* mice had significantly higher fasting glucose levels than *db/*m+ mice. Among *db/db* mice, those treated with CMV‐TIF1γ exhibited lower fasting glucose levels compared with untreated and TGF‐TIF1γ‐treated mice, although this difference was not statistically significant.

### 3.2 TIF1γ Administration Ameliorates Renal Pathology and Dysfunction in *db/db* Mice

**FIGURE 1:**
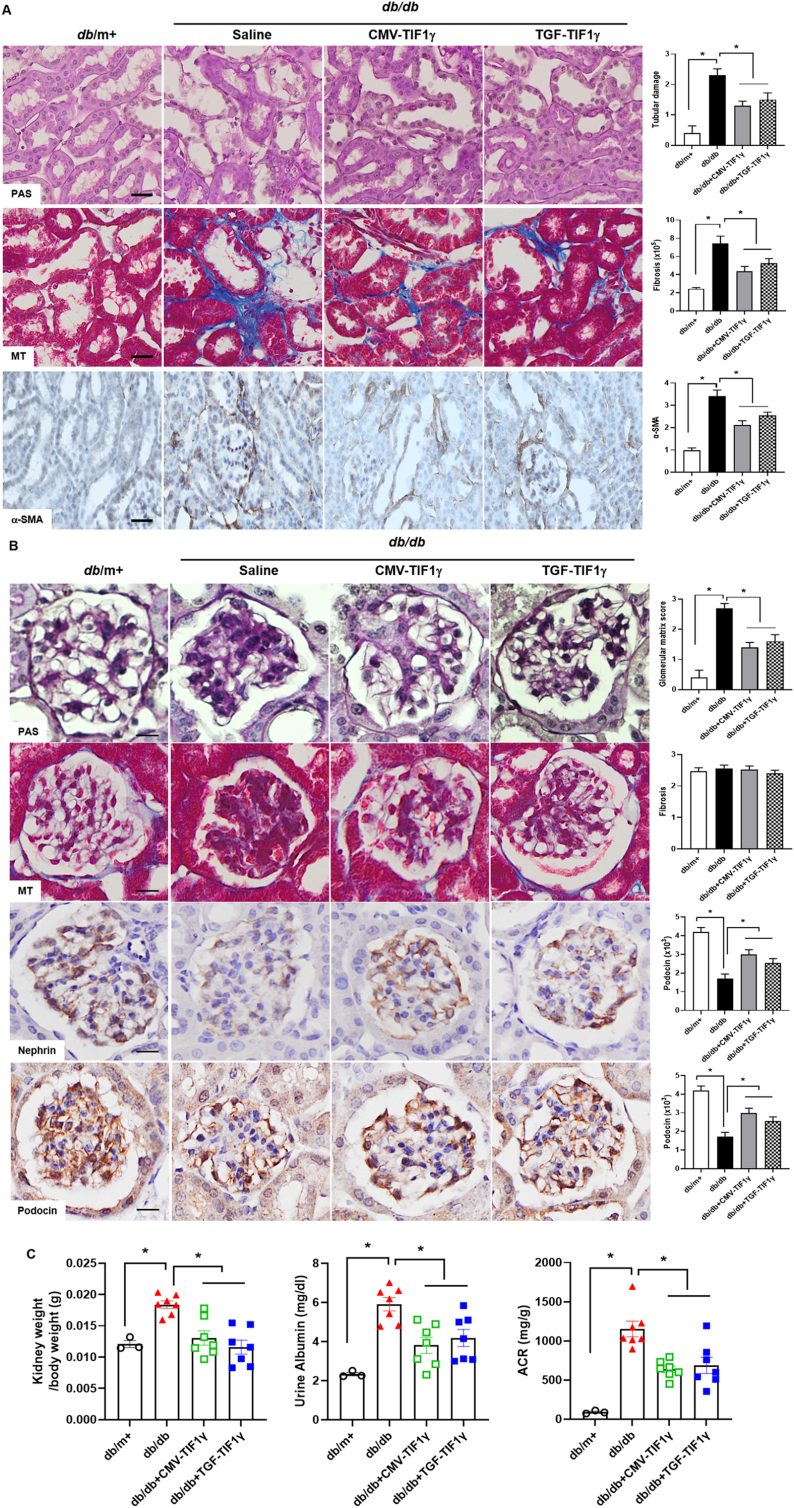
Renal morphology in *db/db* mice treated with or without CMV‐TIF1γ and TGF‐TIF1γ (40 μg/mouse, intraperitoneal). (A) Representative kidney sections stained with PAS and MT, showing tubular and glomerular pathological changes. Tubulointerstitial fibrosis is assessed using α‐SMA immunostaining (α‐SMA in A). Scale bar: 100 μm. (B) Glomerular alterations are evaluated using antinephrin and antipodocin immunostaining (Nephrin and Podocin in B). Scale bar: 50 μm. (C) TIF1γ administration ameliorates kidney function deterioration in *db/db* mice. Kidney weight relative to body weight, urinary albumin levels and the ACR are measured. Statistical significance was determined using a one‐way analysis of variance followed by Tukey's multiple comparison test. Data are presented as mean ± SEM. *p < 0.05. TIF1γ, transcriptional intermediary factor 1γ; TGF, transforming growth factor; CMV, cytomegalovirus; PAS, periodic acid‐Schiff; MT, Masson's trichrome; α‐SMA, alpha‐smooth muscle actin; ACR, albumin‐to‐creatinine ratio; SEM, standard error of the mean.

TIF1γ plasmid administration significantly attenuated renal tubular injury, tubulointerstitial fibrosis and mesangial expansion in *db/db* mice, as evidenced by PAS and MT staining (Figure 1A and B). Untreated *db/db* mice exhibited pronounced renal tubular injury and interstitial fibrosis, whereas both groups of TIF1γ‐treated *db/db* mice showed significantly reduced renal damage (Figure 1A). Immunohistochemical analysis revealed a significant reduction in myofibroblast accumulation, as indicated by α‐SMA‐positive signals in the renal interstitium of both TIF1γ‐treated *db/db* mice, compared with untreated *db/db* mice; this finding suggests that TIF1γ treatment effectively alleviates tubulointerstitial fibrosis (Figure 1A). In addition, TIF1γ treatment improved glomerular mesangial expansion in *db/db* mice (Figure 1B). Nephrin and podocin expression levels, markers of glomerular injury, were significantly elevated in the kidneys of both TIF1γ‐treated *db/db* mice, compared with untreated *db/db* mice (Figure 1B). However, no statistically significant differences were observed in glomerular fibrosis across all groups (Figure 1B). TIF1γ administration influenced kidney weight and function. The kidney weight‐to‐body weight ratio and urinary albumin‐to‐creatinine ratio (ACR) were significantly higher in *db/db* mice compared with *db*/m+ mice. However, these levels were significantly reduced in TIF1γ‐treated *db/db* mice (Figure 1C), emphasizing its protective effects against glomerular and tubular injury in DN.

### 3.3 TIF1γ Administration Suppresses Renal Fibrosis in *db/db* Mice

**FIGURE 2:**
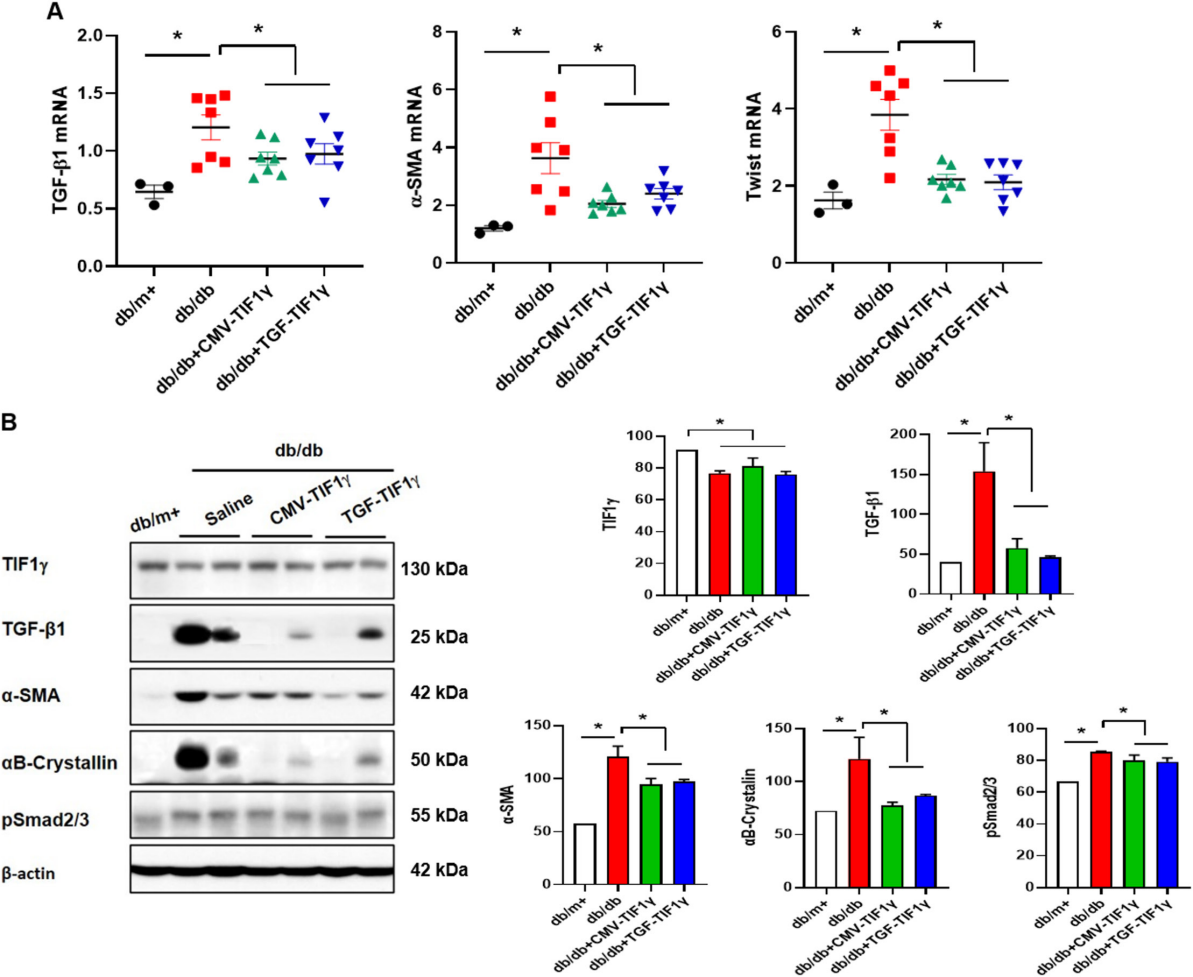
Effect of TIF1γ treatment on EMT markers in *db/db* mice. (A) Effect of TIF1γ plasmid (40 μg) treatment on mRNA expression levels of EMT markers (*TGF‐β1*, *α‐Sma*) and the EMT‐specific transcriptional factor (Twist) were assessed in the kidney using qPCR. (B) Representative immunoblot images and quantitative analysis showing the expression levels of TIF1γ and EMT‐related markers (TGF‐β1, α‐SMA, αβ‐crystalline and pSmad2/3). Statistical significance was determined using a one‐way analysis of variance followed by Tukey's multiple comparison test. Data are presented as mean ± SEM. *p < 0.05. TIF1γ, transcriptional intermediary factor 1γ; EMT, epithelial–mesenchymal transition; TGF‐β1, transforming growth factor beta 1; α‐SMA, alpha‐smooth muscle actin; pSmad2/3, phosphorylated Smad2/3; qPCR, quantitative polymerase chain reaction; mRNA, messenger RNA; SEM, standard error of the mean.

The TGF‐β1/Smad pathway‐mediated EMT plays a crucial role in the development and progression of kidney fibrosis and CKD [17]. In *db/db* mice, the mRNA levels of Tgf‐β1, α‐SMA and Twist, key EMT biomarkers, were significantly higher than those in control *db*/m+ mice. However, TIF1γ plasmid administration significantly reversed these changes (Figure 2A). TIF1γ expression was significantly reduced in the kidneys of *db/db* mice, compared with *db*/m+ mice. However, TIF1γ expression levels did not show a relative increase in *db/db* mice treated with TIF1γ plasmids (Figure 2B). Protein expression patterns of EMT markers mirrored the mRNA findings. The levels of TGF‐β1, pSmad2/3 and profibrotic proteins, such as α‐SMA and αB‐crystallin, were significantly elevated in *db/db* mice. TIF1γ treatment effectively reduced the expression of these proteins (Figure 2B).

### 3.4 Tracing Administered TIF1γ Plasmids In Vivo

**FIGURE 3:**
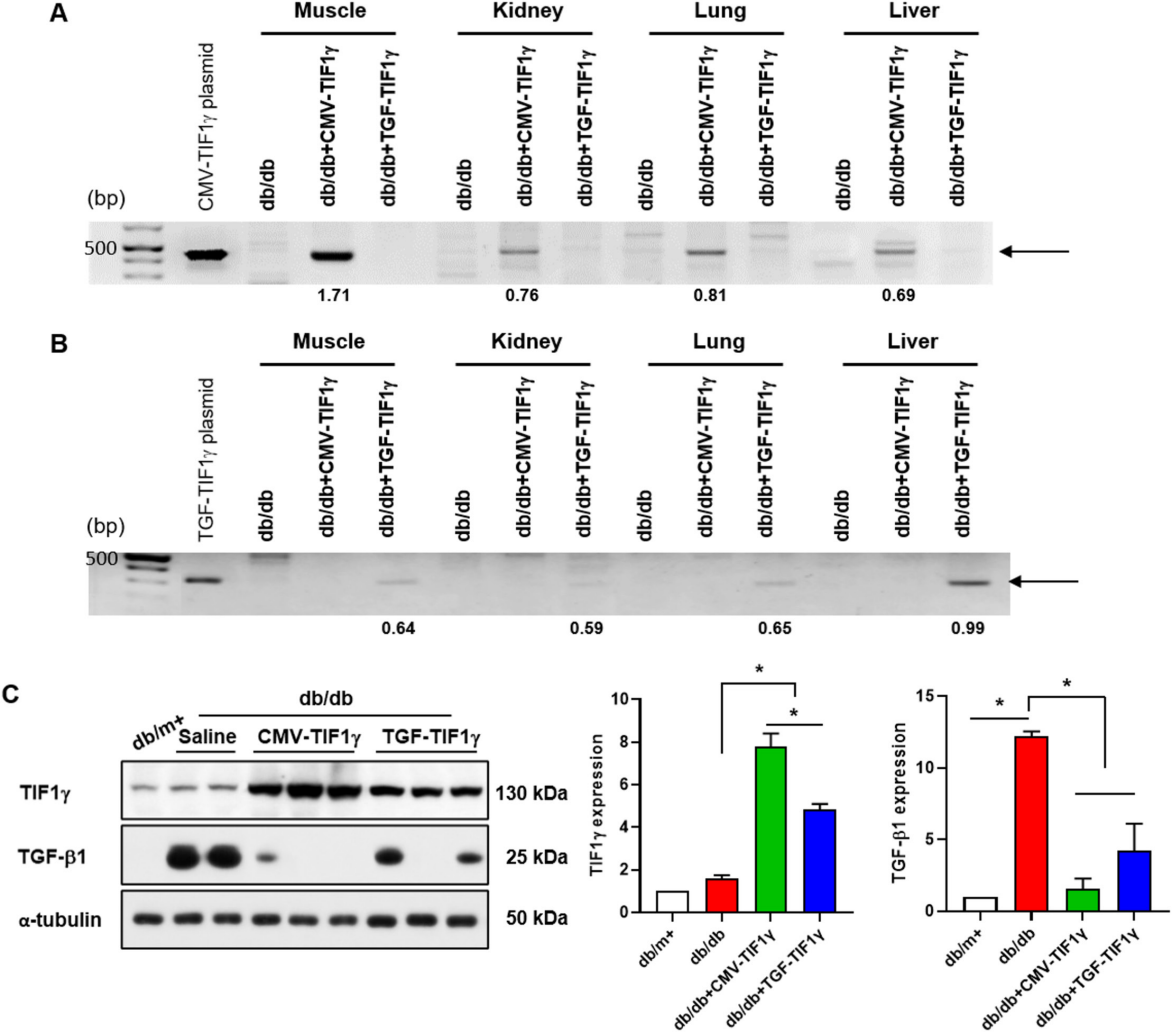
TIF1γ plasmid tracing in various tissues from *db/db mice* using PCR. (A) Representative PCR data using primers targeting the CMV promoter. (B) Representative PCR data using primers targeting the TGF promoter. Effect of TIF1γ treatment (40 μg) on the expression of TIF1γ and TGF‐β1 in the quadriceps muscle of *db/db* mice. (C) Representative immunoblot images and quantitative analysis of TIF1γ and TGF‐β1 expression in quadriceps. Statistical significance was determined using a one‐way analysis of variance followed by Tukey's multiple comparison test. Data are presented as mean ± SEM. *p < 0.05. TIF1γ, transcriptional intermediary factor 1γ; CMV, cytomegalovirus; TGF, transforming growth factor; PCR, polymerase chain reaction; CMV‐ and TGF‐TIF1γ plasmid, 1‐fg plasmid; SEM, standard error of the mean.

Despite plasmid administration, TIF1γ protein expression in *db/db* mice treated with both TIF1γ plasmids did not exhibit a relative increase compared with *db/*m*+* mice (Figure 2B). To confirm the presence of administered TIF1γ plasmids in tissues, we performed genomic PCR and investigated the effects of TIF1γ overexpression on muscle structure and function in *db/db* mice. PCR analysis detected CMV‐ and TGF‐TIF1γ plasmids in the quadriceps muscle, kidney, lungs and liver, with higher levels observed in the quadriceps muscle, lungs and liver, compared with the kidney (Figure 3A and B). Considering this distribution, we focused on plasmid detection in muscle tissue. TIF1γ expression was significantly elevated in the muscles of *db/db* mice treated with both plasmids (Figure 3C). In addition, the increased TGF‐β1 expression indicates that muscle tissues in *db/db* mice provided a favourable environment for the functional activity of TGF‐TIF1γ and CMV‐TIF1γ plasmids (Figure 3C).

### 3.5 TIF1γ Administration Ameliorates Muscle Morphological Changes in *db/db* Mice

**FIGURE 4:**
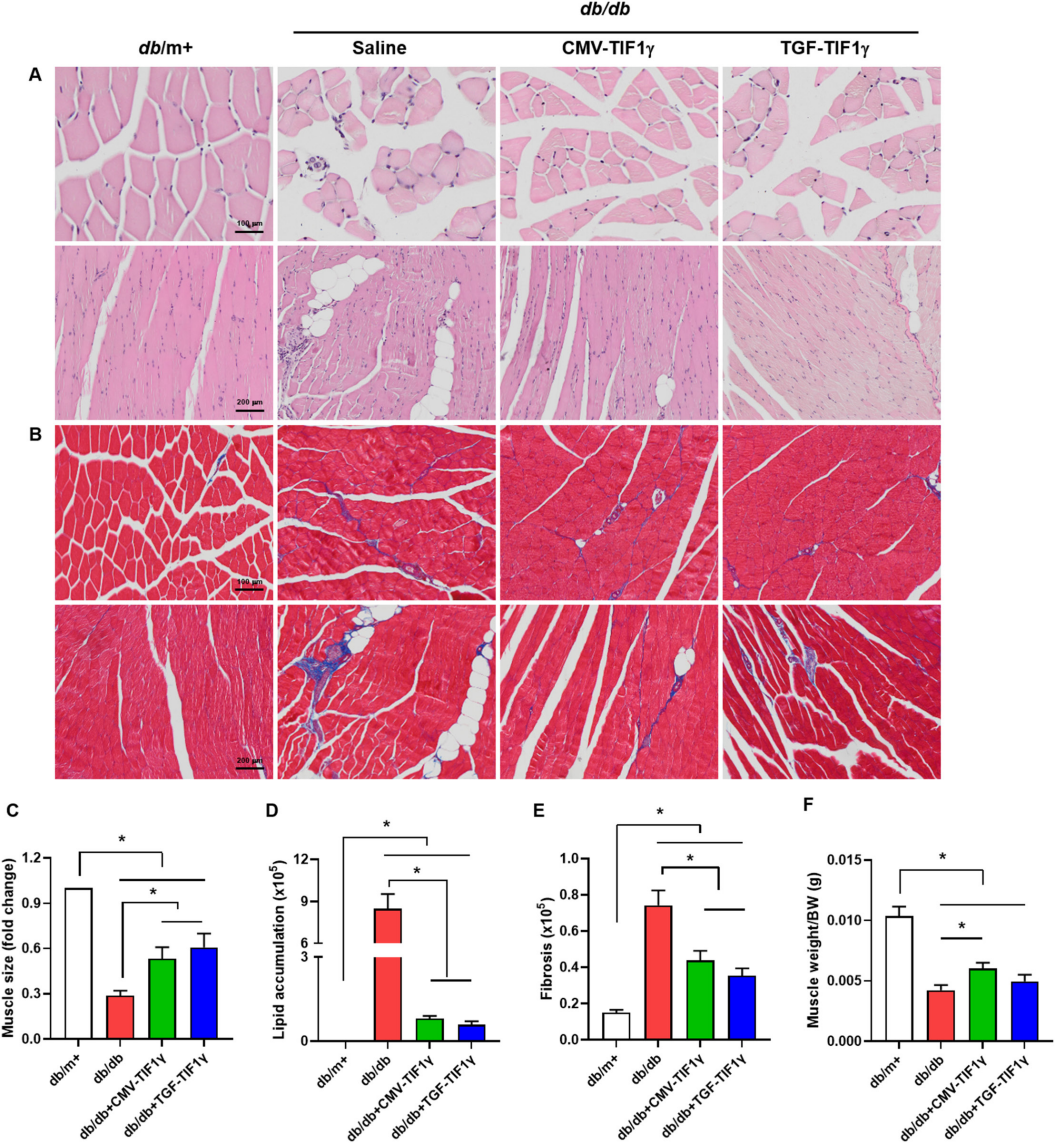
Morphological changes in the quadriceps muscle following TIF1γ treatment. (A & B) Representative H&E and MT staining of quadricep muscles of *db/db* mice including TIF1γ treatment (40 μg). (C) Muscle fibre size, (D) lipid accumulation, (E) fibrosis and (F) muscle weight were assessed based on H&E and MT staining. Statistical significance was determined using a one‐way analysis of variance followed by Tukey's multiple comparison test. Data are presented as mean ± SEM. *p < 0.05 TIF1γ, transcriptional intermediary factor 1γ; MT, Masson's trichrome; H&E, haematoxylin and eosin; SEM, standard error of the mean.

To determine whether increased TIF1γ expression in muscle tissue ameliorates morphological changes and muscle atrophy in *db/db* mice, we isolated and analysed quadriceps muscles using H&E staining. compared with *db*/m+ mice, *db/db* mice exhibited smaller myofibre diameters, irregular myofibre arrangement, increased nuclear density and lipid accumulation. These pathological changes were significantly elevated in *db/db* mice treated with TIF1γ (Figure 4A,C,D). In addition, muscle fibrosis was significantly alleviated in TIF1γ‐treated *db/db* mice, compared with untreated *db/db* mice (Figure 4B,E). CMV‐TIF1γ effectively prevented quadriceps muscle weight loss in *db/db* mice. Although TGF‐TIF1γ treatment also improved muscle loss, the difference between untreated and TGF‐TIF1γ‐treated *db/db* mice was not statistically significant (Figure 4F).

### 3.6 TIF1γ Administration Attenuates Muscle Atrophy and Enhances Muscle Functionality

**FIGURE 5:**
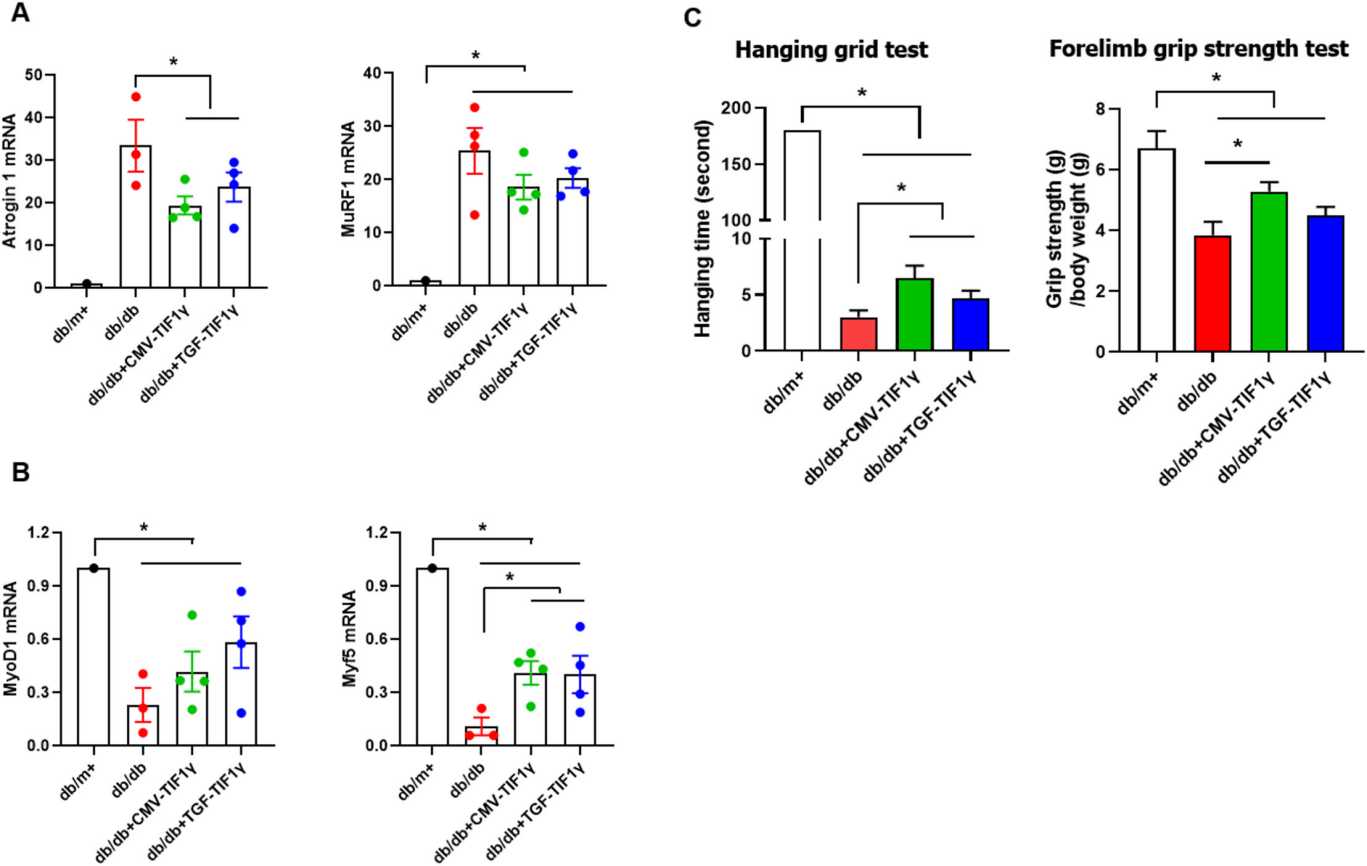
Effect of TIF1γ treatment on gene expression and skeletal muscle function in *db/db* mice. (A) Effect of TIF1γ plasmid (40 μg) treatment on mRNA expression levels of atrophy‐related genes (*Atrogin 1*, *Murf‐1*) and (B) muscle regeneration genes (*Myod1*, *Myf5*) in quadricep muscles. Skeletal muscle functionality was evaluated using the hanging grid and forelimb grip strength tests. Statistical significance was determined using a one‐way analysis of variance followed by Tukey's multiple comparison test. Data are presented as mean ± SEM. *p < 0.05. TIF1γ, transcriptional intermediary factor 1γ; mRNA, messenger RNA; SEM, standard error of the mean.

qRT‐PCR analysis of gene expression revealed the effects of TIF1γ administration on muscle atrophy and regeneration. The expression levels of *Atrogin* 1 and muscle RING‐Finger Protein‐1 (*Murf1*), key markers of muscle atrophy, were significantly higher in *db/db* mice compared with *db/*m+ mice. TIF1γ treatment significantly suppressed the mRNA expression of *Atrogin* 1 and *Murf‐1* in the muscles (Figure 5A). Conversely, the mRNA levels of muscle regeneration‐related genes, *Myod1* and *Myf5*, were significantly reduced in *db/db* mice; this reduction was effectively reversed by TIF1γ treatments (Figure 5B). To evaluate muscle functionality, we conducted hanging grid and forelimb grip strength tests. Both tests demonstrated significantly weaker performance in *db/db* mice than in *db/*m+ mice. However, TIF1γ‐treated *db/db* mice exhibited significant improvements in muscle strength and endurance relative to untreated *db/db* mice (Figure 5C).

### 3.7 TIF1γ Administration Enhances Myokine Levels in Muscle Tissues of *db/db* Mice

**FIGURE 6:**
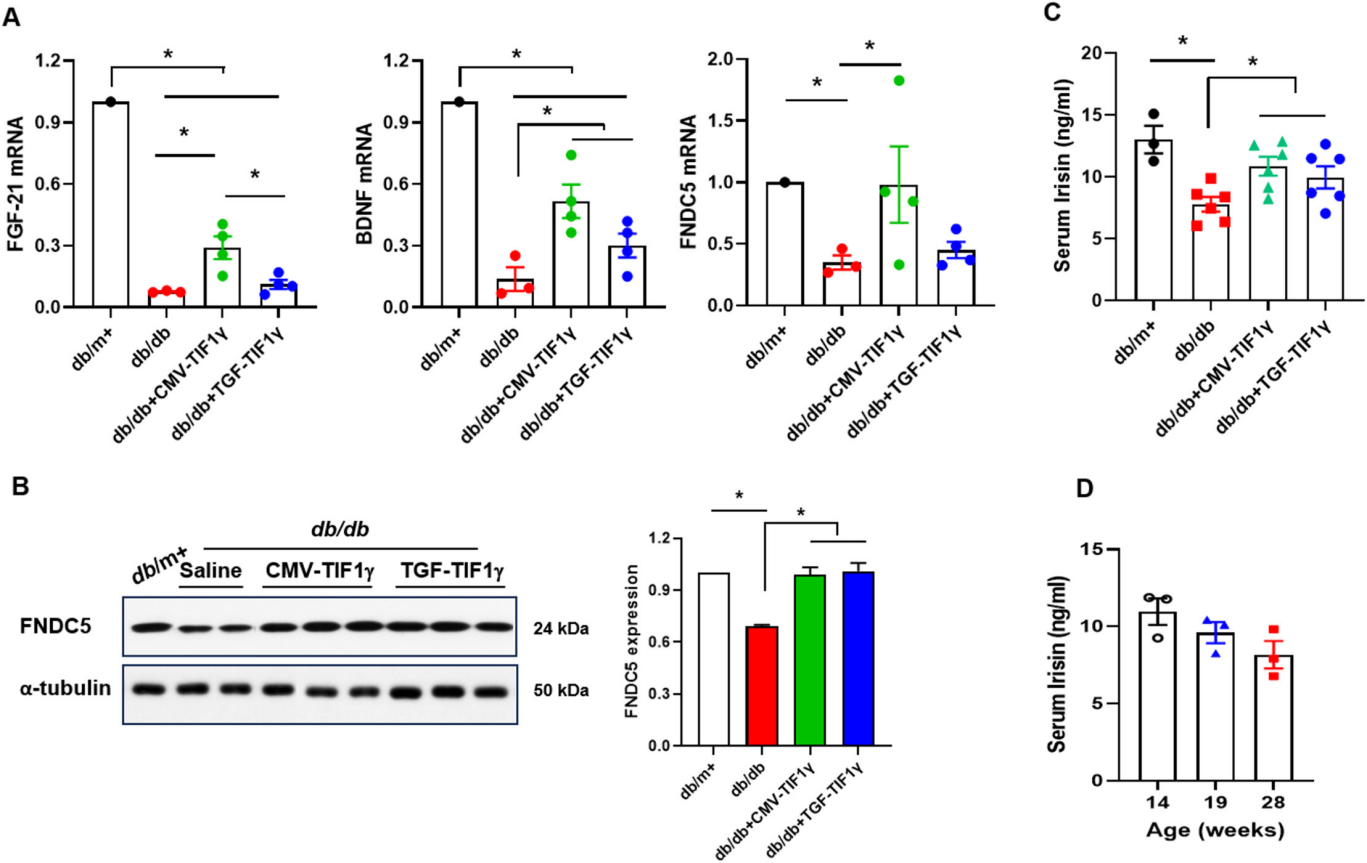
Effect of TIF1γ treatment on myokine expression in the quadricep muscles of *db/db* mice. (A) mRNA expression levels of the myokines *FGF‐21*, *BDNF* and *FNDC5* were analysed using qPCR. (B) Representative immunoblot images and quantitative analysis of FNDC5 protein expression. (C) Effect of TIF1γ plasmid (40 μg) treatment on serum irisin levels, measured using an ELISA. (D) Changes in serum irisin levels over time in normal male C57BL/6 mice. Statistical significance was determined using a one‐way analysis of variance followed by Tukey's multiple comparison test. Data are presented as mean ± SEM. *p < 0.05. TIF1γ, transcriptional intermediary factor 1γ; qPCR, quantitative polymerase chain reaction; Fgf‐21, fibroblast growth factor 21; BDNF, brain‐derived neurotrophic factor; SEM, standard error of the mean; mRNA, messenger RNA.

Myokines, secreted by skeletal muscle, play crucial roles in autocrine regulation of muscle metabolism and influence the metabolic functions of other tissues and organs [18, 19]. In *db/db* mice, the mRNA expression levels of FGF‐21, BDNF and FNDC5 in the quadriceps muscle were significantly reduced, compared with those in *db/*m+ mice. However, TIF1γ administration effectively mitigated this downregulation in *db/db* mice (Figure 6A). Numerous studies have demonstrated that irisin levels are affected by metabolic diseases and other pathological conditions [19, 20, 21]. Consistent with the decreased FNDC5 mRNA levels, reduction in FNDC5 protein levels was observed in the muscles of *db/db* mice (Figure 6B). Serum irisin levels were significantly lower in *db/db* mice than in *db/*m+ mice and TIF1γ treatment significantly restored these levels (Figure 6C). Notably, serum irisin levels were observed to decline with age in mice (Figure 6D). In addition, we observed a reduction in BDNF and FGF‐21 protein levels in the muscles, which were significantly reversed by TIF1γ administration (Figure S2A).

### 3.8 TIF1γ Transfection Modulates Irisin Expression and Secretion in C2C12 Cells and Alters EMT in HK‐2 Cells

**FIGURE 7:**
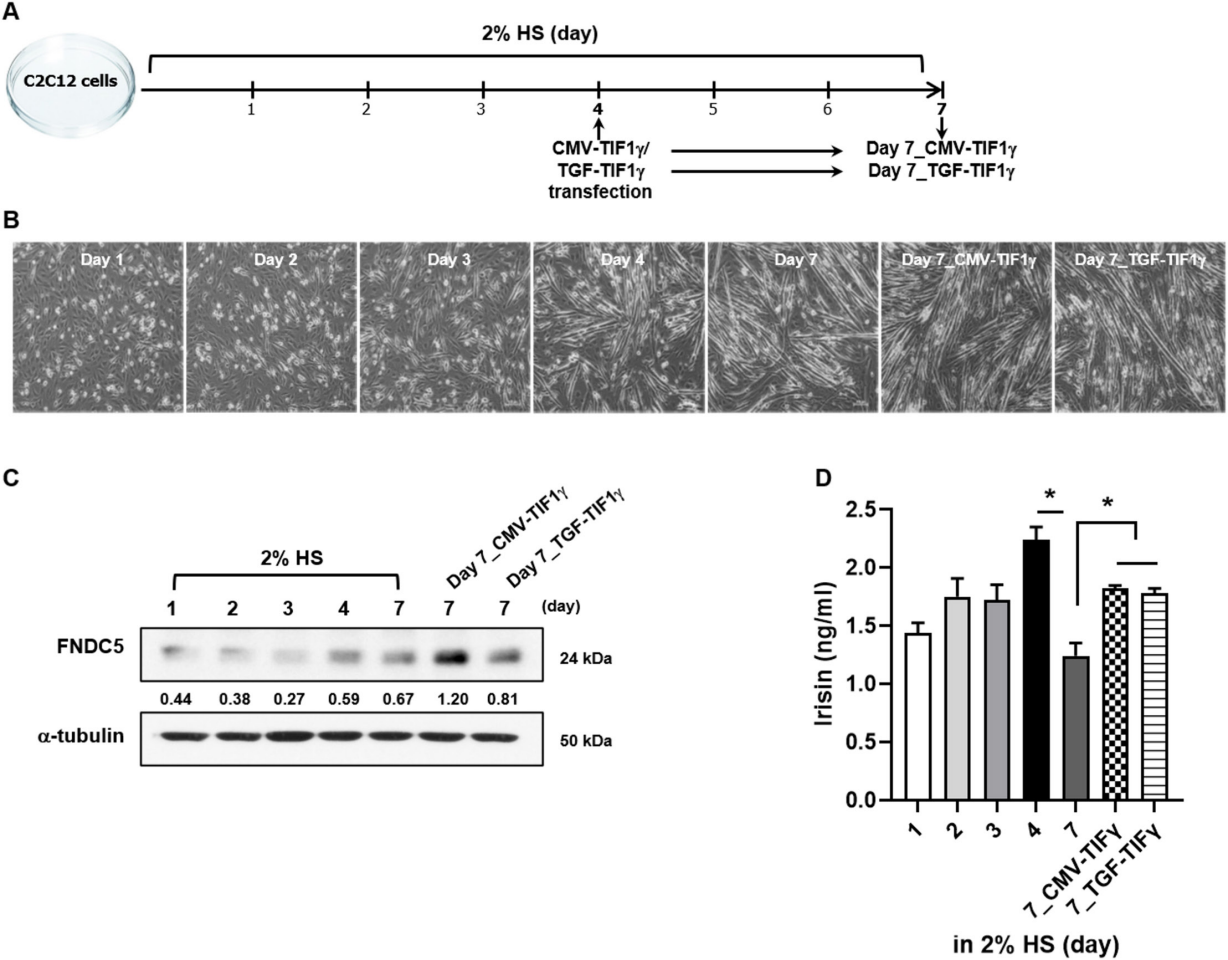
Effect of TIF1γ on irisin expression and secretion in C2C12 cells. (A & B) Experimental in vitro scheme for C2C12 differentiation. (C & D) Changes in irisin expression and secretion following TIF1γ plasmid (2 μg) transfection during C2C12 differentiation. Statistical significance was determined using a one‐way analysis of variance followed by Tukey's multiple comparison test. Data are presented as mean ± SEM. *p < 0.05. TIF1γ, transcriptional intermediary factor 1γ; C2C12, murine myoblast cells; SEM, standard error of the mean.

**FIGURE 8:**
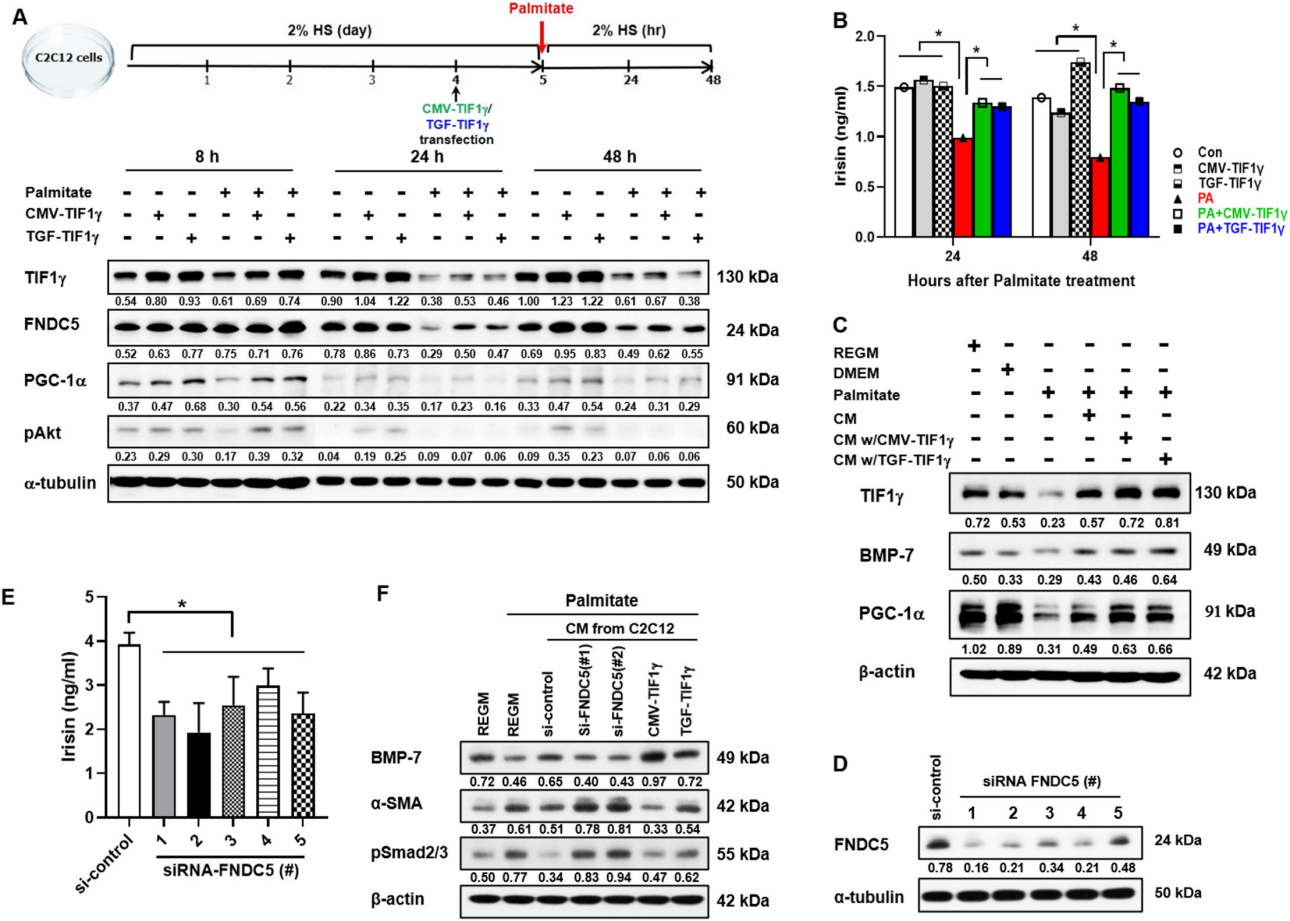
Effect of TIF1γ on palmitate treatment in C2C12 cells and the paracrine effect of TIF1γ‐induced irisin on palmitate‐treated HK‐2 cells. (A) Changes in irisin, TIF1γ, PGC‐1α and pAkt expression following palmitate (100 μM) treatment in the presence or absence of TIF1γ (2 μg). (B) Changes in irisin secretion following palmitate (100 μM) treatment with or without TIF1γ (2 μg). (C) Changes in EMT markers in palmitate (100 μM)‐treated HK‐2 cells induced by CM from TIF1γ‐transfected C2C12 cells. (D) Western blot analysis of FNDC5 protein levels in C2C12 cells transfected with control or FNDC5‐specific siRNAs (#1–5) for 48 h. (E) Irisin levels in CM from C2C12 cells transfected with 25 nmol siRNA‐FNDC5 and 2 μg TIF1γ, measured by ELISA. (F) Changes in EMT markers in palmitate (100 μM)‐treated HK‐2 cells induced by CM from C2C12 cells transfected with 25 nmol siRNA‐FNDC5 and TIF1γ. Each protein expression level was normalized to α‐tubulin or β‐actin (A, C, D, F). TIF1γ, transcriptional intermediary factor 1γ; HK‐2, human kidney 2 cells; C2C12, murine myoblast cells; PGC‐1α, peroxisome proliferator‐activated receptor gamma coactivator 1‐alpha; pAkt, phosphorylated protein kinase B; EMT, epithelial–mesenchymal transition; BMP‐7, bone morphogenetic protein 7; α‐SMA, alpha‐smooth muscle actin; pSmad2/3, phosphorylated Smad2/3; CM, conditioned medium; siRNA, signal interfering RNA.

To elucidate the direct involvement of TIF1γ overexpression in irisin expression and secretion, C2C12 myoblasts were used. Cellular morphological alterations under differentiation conditions were assessed, revealing no significant disparities following TIF1γ transfection (Figure 7A,B). Notably, irisin protein expression and secretion exhibited a peak at 4 days post‐differentiation, followed by a subsequent decline at day 7. However, TIF1γ plasmid transfection at day 7 significantly augmented irisin levels (Figure 7C,D). To simulate in vivo conditions, C2C12 cells were exposed to palmitate, a saturated fatty acid that represents elevated circulating free fatty acids in an obese environment, with or without TIF1γ transfection (Figure 8A). Palmitate treatment resulted in a reduction in irisin and TIF1γ expression, whereas TIF1γ transfection mitigated these effects. Furthermore, palmitate exposure led to decreased PGC‐1α and pAkt expression in C2C12 cells, effects that were reversed by TIF1γ transfection (Figure 8A). Concomitantly, the concentration of irisin in the culture medium was significantly decreased in palmitate‐treated C2C12 myoblasts, a phenomenon that was reversed by TIF1γ transfection (Figure 8B). To assess the role of TIF1γ in muscle‐kidney crosstalk, palmitate‐treated HK‐2 cells, were incubated with conditioned medium from non‐transfected and TIF1γ‐transfected C2C12 cells. The protein expression levels of BMP‐7, an epithelial phenotype marker, and PGC‐1α were reduced in palmitate‐treated HK‐2 cells cultured in a conditioned medium from non‐transfected C2C12 cells. However, these levels were restored when the conditioned medium was derived from TIF1γ‐transfected C2C12 cells (Figure 8C). Previous studies have reported that the myokine, irisin, mediates muscle‐kidney crosstalk, providing protective effects against kidney damage [22]. To confirm whether TIF1γ influences irisin‐mediated kidney protection, FNDC5 knockdown was performed using siRNA in C2C12 cells, followed by an evaluation of its effects on palmitate‐treated HK‐2 cells and EMT regulation. Immunoblot and ELISA analyses confirmed the effective knockdown of FNDC5 (Figure 8D and E). Our findings demonstrated that conditioned medium from FNDC5‐knockdown C2C12 cells accelerated EMT in palmitate‐treated HK‐2 cells, as indicated by decreased BMP‐7 expression and increased levels of α‐SMA and pSmad2/3, compared with palmitate treatment alone and siRNA controls (Figure 8F). In contrast, conditioned medium from TIF1γ‐overexpressing C2C12 cells abolished palmitate‐induced EMT (Figure 8F). In addition, to assess the potential involvement of other myokines in EMT regulation, BDNF and FGF‐21 knockdowns were performed in C2C12 cells. When palmitate‐treated HK‐2 cells were incubated in a conditioned medium from BDNF‐ or FGF‐21‐knockdown C2C12 cells, EMT was not accelerated (Figure S2C and E). This outcome differed from the FNDC5 knockdown results. These findings suggest that TIF1γ overexpression contributes to the restoration of myokine levels, including BDNF, FGF‐21 and irisin, under metabolic stress. However, irisin specifically mediates the protective effects of TIF1γ overexpression on renal cells.

## 4 Discussion

We investigated the therapeutic potential of TIF1γ in mitigating DN‐induced complications in the kidneys and skeletal muscles using *db/db* mice. Our findings revealed that TIF1γ administration significantly alleviated kidney dysfunction, renal tubulointerstitial fibrosis and glomerular mesangial expansion. In addition, TIF1γ preserved muscle weight and functionality while attenuating muscle atrophy in *db/db* mice, emphasizing its multiorgan protective effects in DM.

Although TIF1γ‐plasmid overexpression exhibited a trend toward improvements in body weight and blood glucose levels, these changes were not statistically significant, indicating that TIF1γ may not directly influence glucose regulation. Although hyperglycaemia is widely regarded as a primary driver of diabetic kidney disease (DKD) progression, recent studies indicate that it may not be the sole etiological factor [23, 24]. For instance, Song et al. [25] reported that Sestrin2, despite its efficacy in preventing glomerular manifestations in diabetic mice, did not significantly lower blood glucose levels, consistent with our findings.

The progression of DN is closely associated with CKD, with TGF‐β1–induced fibrosis serving as a hallmark of renal injury and dysfunction [7, 26, 27]. Consistent with prior studies, untreated *db/db* mice exhibited substantial renal fibrosis, characterized by increased expression of α‐SMA and other profibrotic markers, including TGF‐β1 and pSmad2/3, indicative of active EMT [28]. However, TIF1γ administration resulted in a significant reduction in the expression of these pro‐fibrotic factors, significantly mitigating renal fibrosis and dysfunction. The observed reduction in glomerular and tubular damage following TIF1γ treatment aligns with its established role as a TGF‐β1 pathway inhibitor, indicating that TIF1γ may attenuate renal EMT and fibrosis by interfering with TGF‐β1 signalling.

The integrity of skeletal muscle is frequently compromised in DN patients, exacerbating treatment challenges and impacting overall prognosis [19, 20, 21, 22]. We observed that TIF1γ treatment alleviated muscle fibre atrophy, lipid accumulation and fibrosis in *db/db* mice while improving muscle functionality. These benefits may be attributed to TIF1γ‐induced alterations in muscle gene expression, particularly the upregulation of genes associated with muscle regeneration. By preserving muscle integrity and function, TIF1γ may contribute to the amelioration of systemic metabolic dysfunction in diabetic conditions.

We observed that TIF1γ plasmids were more abundant in skeletal muscle than in renal tissues, yet therapeutic benefits were evident in both organs. This effect may be attributed to muscle‐kidney crosstalk mediated by myokines, such as irisin, released from TIF1γ‐expressing myocytes. Supporting this hypothesis, our in vitro findings demonstrated that conditioned media derived from TIF1γ‐transfected C2C12 cells, enriched in irisin, effectively reduced EMT markers in palmitate‐stressed HK‐2 cells, indicating that soluble factors secreted by muscle cells can exert protective effects on renal cells. These findings suggest a potential mechanism wherein TIF1γ expression in muscle indirectly enhances renal health through myokine‐mediated signalling, emphasizing a promising dual‐organ therapeutic strategy.

Skeletal muscle functions as an endocrine organ, secreting various myokines, including irisin, BDNF, FGF‐21, myonectin, musclin, interleukin (IL)‐6 and IL‐13, all of which regulate the metabolic functions of multiple tissues and organs [18]. Notably, skeletal muscle insulin resistance plays a critical role in the pathogenesis of T2DM [29]. Irisin, a myokine secreted from skeletal muscle in a PGC‐1α‐dependent manner during exercise, is the proteolytically cleaved form of FNDC5 and has been implicated in lowering blood glucose levels and improving insulin resistance [30]. Reduced FNDC5 expression has been reported in patients with T2DM and *db/db* mice [31, 32]. Considering its role in regulating metabolic homeostasis, irisin has garnered significant attention due to its broad pathophysiological roles in metabolic disorders [33]. It exhibits antioxidant, anti‐inflammatory and antiapoptotic properties, making it a promising therapeutic and diagnostic target for conditions, such as DM, obesity, nonalcoholic fatty liver disease, osteoporosis and cancer [33, 34, 35]. We observed lower serum irisin levels in individuals with insulin resistance and T2DM, which may provide new insights into the pathology of these conditions. Similarly, reduced serum irisin levels were observed in *db/db* mice. Previous studies have demonstrated significantly lower irisin levels in individuals with T2DM compared with controls, irrespective of age, sex or body mass index [36]. In patients with DM, vascular complications resulting from endothelial dysfunction are a major cause of mortality [37]. Notably, irisin partially alleviates endothelial dysfunction in T2DM by reducing oxidative and nitrative stress through the inhibition of NF‐κB/iNOS and PKC‐α/NADPH oxidase signalling pathways [37]. Collectively, these findings suggest that decreased serum irisin levels may contribute to the progression of CKD, emphasizing the urgent need for novel therapeutic strategies targeting both skeletal muscle and kidney function in CKD patients.

PGC‐1α expression, predominantly in skeletal muscle, plays a crucial role in regulating various metabolic parameters, including irisin production [38]. Irisin has been shown to mediate diverse biological functions, including adipocyte browning, neural differentiation, osteoblast proliferation, myogenic differentiation and myoblast fusion, primarily through intracellular signalling pathways such as the mitogen‐activated protein kinase signalling cascade [19]. In addition, other signalling cascades, such as the AMP‐activated protein kinase, phosphatidylinositol 3‐kinase/protein kinase B and signal transducer and activator of transcription 3/Snail pathways, mediate critical functions of FNDC5/Irisin [39]. Our data revealed that palmitate treatment reduced the expression levels of irisin, TIF1γ, PGC‐1α and pAkt in C2C12 cells, an effect that was reversed by TIF1γ transfection. Notably, the upregulation of PGC‐1α and pAkt protein expression following TIF1γ transfection preceded the increase in FNDC5 protein expression in C2C12 cells. These findings suggest that the PGC‐1α and AKT pathways may play a role in TIF1γ‐mediated FNDC5 expression and secretion in muscle cells. However, the precise mechanisms underlying irisin signal transduction in response to TIF1γ remain uncertain.

Our study has several limitations. First, we did not establish a direct association between TIF1γ administration and fat loss in vivo. Further studies are needed to investigate the potential causal relationship between TIF1γ and impaired irisin signalling in adipose tissue and skeletal muscle, as well as its role in the development of T2DM. Second, we exclusively analysed the quadriceps muscle, excluding other muscles such as the gastrocnemius and soleus. This decision was driven by the technical challenges associated with harvesting these muscles from 25‐week‐old *db/db* mice and the need to prevent data bias from fat contamination. Third, we did not establish a correlation between muscle functionality, serum irisin levels and the pathology of CKD or kidney function in patients with DM and obesity‐associated CKD.

In summary, our findings emphasize the potential of TIF1γ as a multitargeted therapeutic agent for DN, mitigating both renal and muscular complications through direct fibrosis inhibition and indirect myokine‐mediated inter‐organ crosstalk. However, considering the exploratory nature of our study, further studies are needed to elucidate the underlying molecular pathways and validate these findings in human models before TIF1γ can be considered for clinical application as a dual‐target therapy for DKD.

## Ethics Statement

The Institutional Ethics Committee of the Gyeongsang National University Institutional Animal Care and Gyeongsang National University approved the study protocols (GNU‐210202‐M0011‐01). The study was performed in accordance with the regulations and guidelines established by this committee.

## Conflicts of Interest

The authors declare no conflicts of interest.

## Supporting information


**Figure S1** Experimental scheme and effects of TIF1γ administration in *db/db* mice with type 2 diabetes mellitus. (A) Schematic representation of the experimental design to evaluate the effects of TIF1γ in db/db mice. CMV‐TIF1γ and TGF‐TIF1γ plasmids (40 μg/mouse) were intraperitoneally administered once weekly for 16 weeks. (B & C) Effects of TIF1γ administration on body weight and serum glucose level. Further details are provided in the Materials and Methods section. TIF1γ, transcriptional intermediary factor 1γ; CMV, cytomegalovirus; TGF, transforming growth factor.

**Figure S2** Effect of TIF1γ treatment on myokine expression and paracrine effects of TIF1γ‐induced BDNF and FGF‐21 on palmitate‐treated HK‐2 cells. (A) Representative immunoblot images and quantitative analysis of BDNF and FGF‐21 protein levels in the quadriceps muscle of db/db mice following weekly intraperitoneal administration of CMV‐TIF1γ and TGF‐TIF1γ plasmids (40 μg/mouse) for 16 weeks. (B & D) Western blot analysis of BDNF and FGF‐21 protein levels in C2C12 cells transfected with control or 25 nmol of siRNAs specific for BDNF and FGF‐21 (#1–4) for 48 h. (C & E) Representative images and quantitative analysis of EMT markers in palmitate (100 μM)‐treated HK‐2 cells exposed to conditioned medium from C2C12 cells transfected with 25 nmol of siRNA‐BDNF, siRNA‐FGF‐21 (#2 and 3) and 2 μg TIF1γ. Each protein expression level was normalized to α‐tubulin or β‐actin. Statistical significance was determined using a one‐way analysis of variance followed by Tukey’s multiple comparison test. Data are presented as mean ± SEM. *p < 0.05.

**Data S1** Supplementary Information.
