# Supplementary figures and images for: Transcriptional Intermediary Factor 1γ–Induced Irisin in Skeletal Muscle Attenuates Renal Fibrosis in Diabetic Nephropathy
Source: J Cachexia Sarcopenia Muscle. 2025 Apr 15;16(2):e13810. doi: 10.1002/jcsm.13810 (PMC12000539; doi:10.1002/jcsm.13810)

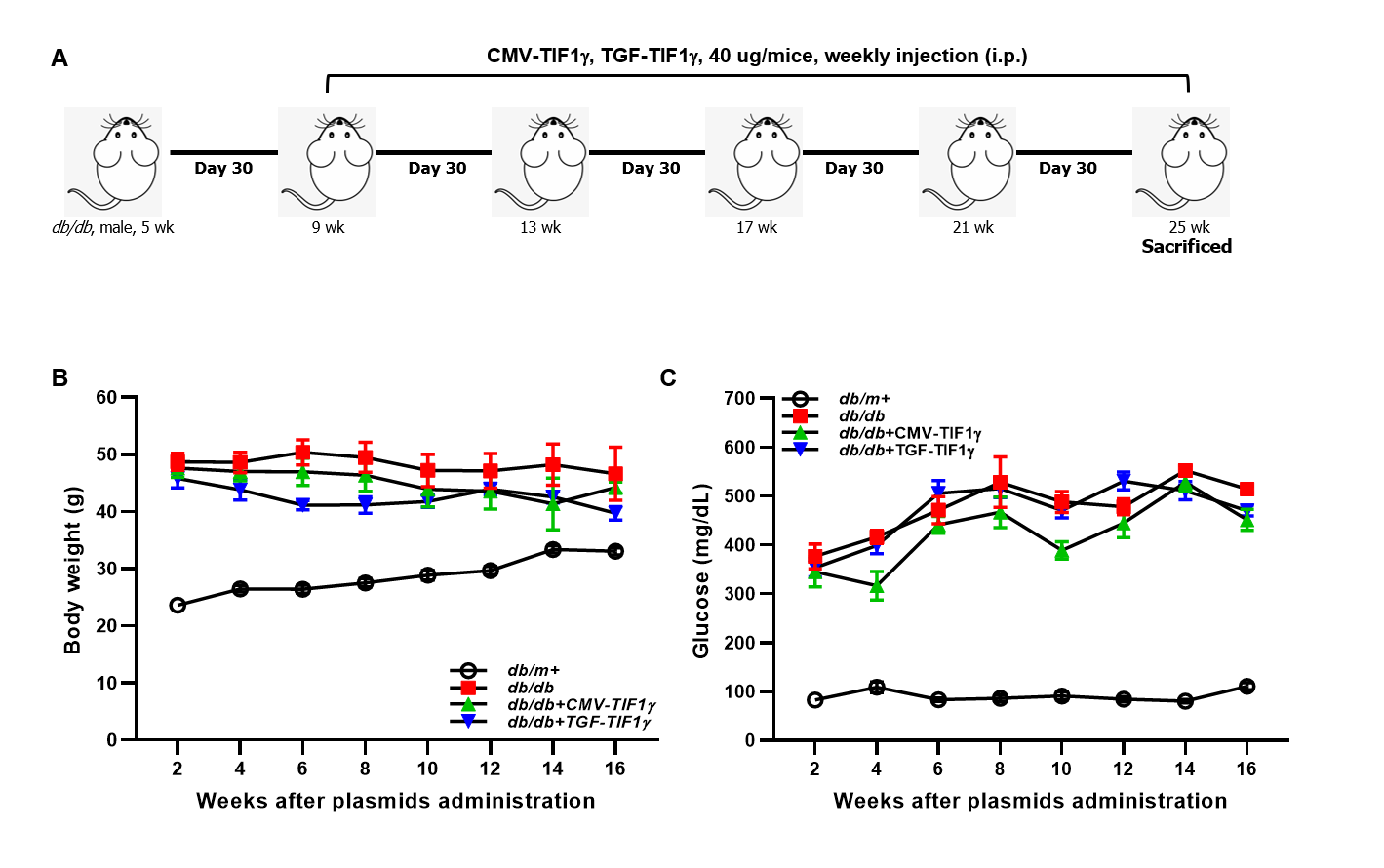

Supplement: Supplementary file 1 — Figure S1 Experimental scheme and effects of TIF1γ administration in db/db mice with type 2 diabetes mellitus. (A) Schematic representation of the experimental design to evaluate the effects of TIF1γ in db/db mice. CMV‐TIF1γ and TGF‐TIF1γ plasmids (40 μg/mouse) were intraperitoneally administered once weekly for 16 weeks. (B & C) Effects of TIF1γ administration on body weight and serum glucose level. Further details are provided in the Materials and Methods section. TIF1γ, transcriptional intermediary factor 1γ; CMV, cytomegalovirus; TGF, transforming growth factor. [file JCSM-16-e13810-s002.tif]

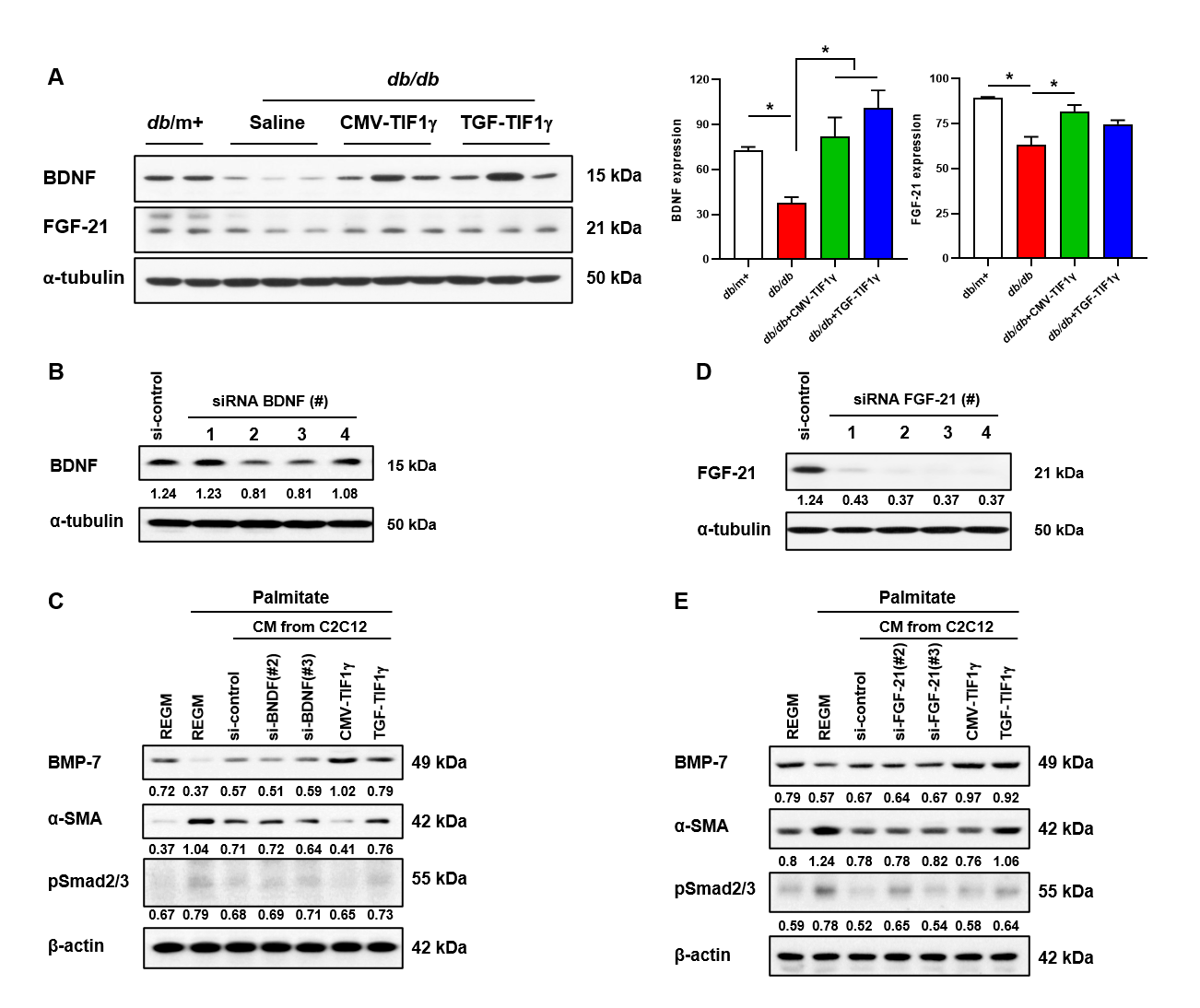

Supplement: Supplementary file 2 — Figure S2 Effect of TIF1γ treatment on myokine expression and paracrine effects of TIF1γ‐induced BDNF and FGF‐21 on palmitate‐treated HK‐2 cells. (A) Representative immunoblot images and quantitative analysis of BDNF and FGF‐21 protein levels in the quadriceps muscle of db/db mice following weekly intraperitoneal administration of CMV‐TIF1γ and TGF‐TIF1γ plasmids (40 μg/mouse) for 16 weeks. (B & D) Western blot analysis of BDNF and FGF‐21 protein levels in C2C12 cells transfected with control or 25 nmol of siRNAs specific for BDNF and FGF‐21 (#1–4) for 48 h. (C & E) Representative images and quantitative analysis of EMT markers in palmitate (100 μM)‐treated HK‐2 cells exposed to conditioned medium from C2C12 cells transfected with 25 nmol of siRNA‐BDNF, siRNA‐FGF‐21 (#2 and 3) and 2 μg TIF1γ. Each protein expression level was normalized to α‐tubulin or β‐actin. Statistical significance was determined using a one‐way analysis of variance followed by Tukey’s multiple comparison test. Data are presented as mean ± SEM. *p < 0.05. [file JCSM-16-e13810-s001.tif]
