# Supplementary material for: Transcriptional Intermediary Factor 1γ–Induced Irisin in Skeletal Muscle Attenuates Renal Fibrosis in Diabetic Nephropathy
Source: J Cachexia Sarcopenia Muscle. 2025 Apr 15;16(2):e13810. doi: 10.1002/jcsm.13810 (PMC12000539; doi:10.1002/jcsm.13810)
Supplement: Supplementary file 3 — Data S1 Supplementary Information. [file JCSM-16-e13810-s003.docx]

**Transcriptional Intermediary Factor 1γ-Induced Irisin in Skeletal Muscle Attenuates Renal Fibrosis in Diabetic Nephropathy**

Jin Hyun Kim^1,2†^, Seunghye Lee^3†^, Hani Jang^2,3^, Sehyun Jung^3^, Myeong Hee Jung^1^, Jeong Won Yun^1^, Haejin Jeon^3^, Hyun-Jung Kim^2,3,^*, Se-Ho Chang ^2,3^*, Eun Ju Lee^4^, Hyo-Soo Kim^5,6^

^1^ Biomedical Research Institute, Gyeongsang National University Hospital, Jinju 52727, Republic of Korea.

^2^ Institute of Medical Science, Gyeongsang National University, Jinju 52727, Republic of Korea.

^3^ Division of Nephrology, Department of Internal Medicine, Gyeongsang National University College of Medicine and Gyeongsang National University Hospital, Jinju 52727, Republic of Korea.

^4^ Biomedical Research Institute, Seoul National University Hospital, Seoul 03080, Republic of Korea.

^5^ Molecular Medicine & Biopharmaceutical Sciences, Seoul National University, Seoul 03080, Republic of Korea.

^6^ Department of Internal Medicine, Seoul National University College of Medicine, Seoul 03080, Republic of Korea.

^†^ These authors contributed equally to this work.

***** Correspondence:

Se-Ho Chang,

Tel. +82-55-750-8067

Fax: +82-55-755-9078

[shchang@gnu.ac.kr](mailto:shchang@gnu.ac.kr)

Hyun-Jung Kim

Tel.: +82-55-750-8875

Fax: +82-55-755-9078

[kimhjyh@gnu.ac.kr](mailto:kimhjyh@gnu.ac.kr)


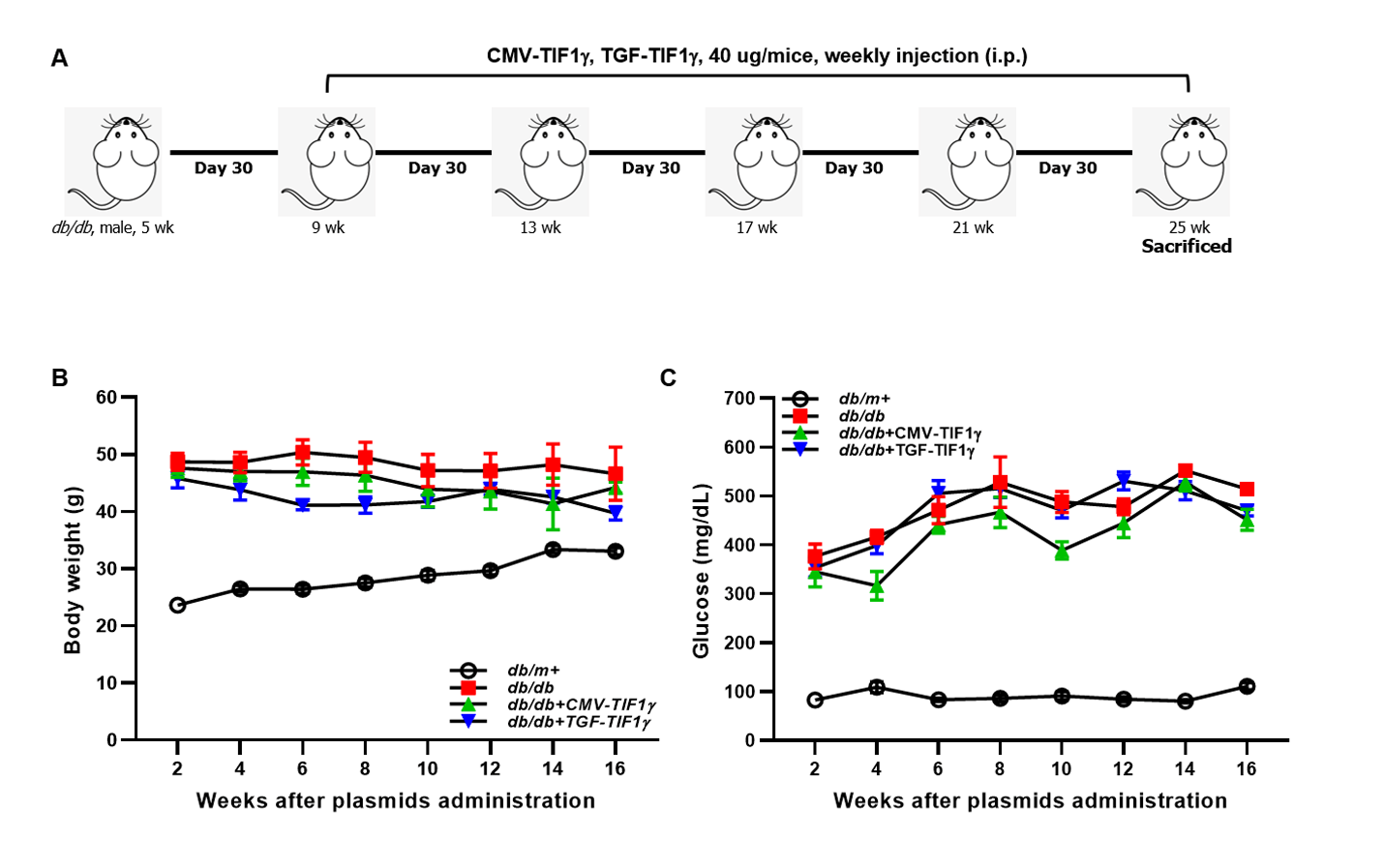


**Fig.** S**1** Experimental scheme and effects of TIF1γ administration in *db/db* mice with type 2 diabetes mellitus. (A) Schematic representation of the experimental design to evaluate the effects of TIF1γ in db/db mice. CMV-TIF1γ and TGF-TIF1γ plasmids (40 μg/mouse) were intraperitoneally administered once weekly for 16 weeks. (B & C) Effects of TIF1γ administration on body weight and serum glucose level. Further details are provided in the Materials and Methods section.

TIF1γ, transcriptional intermediary factor 1γ; CMV, cytomegalovirus; TGF, transforming growth factor.

**
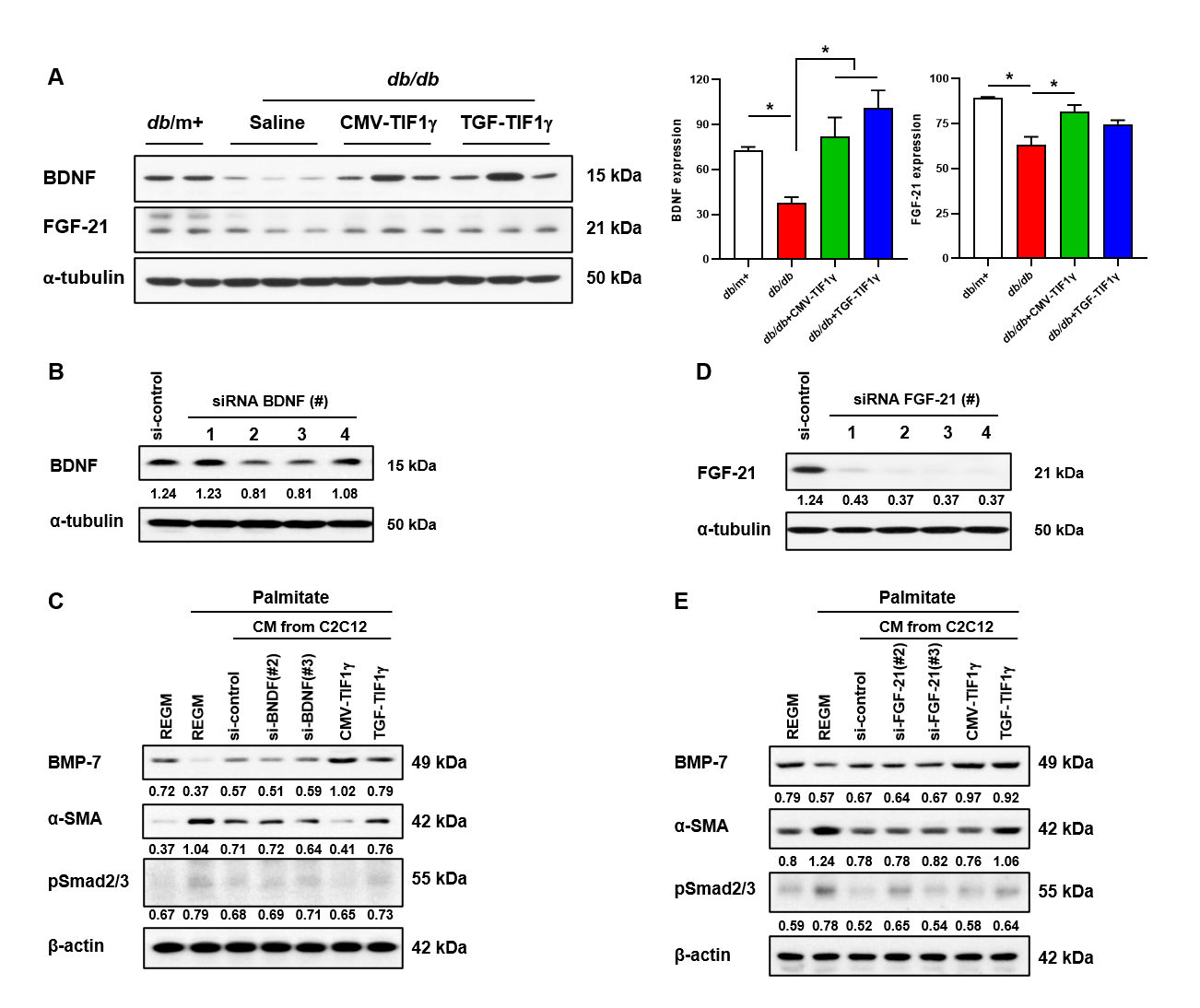
**

**Fig**. S2 Effect of TIF1γ treatment on myokine expression and paracrine effects of TIF1γ-induced BDNF and FGF-21 on palmitate-treated HK-2 cells. (A) Representative immunoblot images and quantitative analysis of BDNF and FGF-21 protein levels in the quadriceps muscle of db/db mice following weekly intraperitoneal administration of CMV-TIF1γ and TGF-TIF1γ plasmids (40 μg/mouse) for 16 weeks. (B & D) Western blot analysis of BDNF and FGF-21 protein levels in C2C12 cells transfected with control or 25 nmol of siRNAs specific for BDNF and FGF-21 (#1-4) for 48 h. (C & E) Representative images and quantitative analysis of EMT markers in palmitate (100 μM)-treated HK-2 cells exposed to conditioned medium from C2C12 cells transfected with 25 nmol of siRNA-BDNF, siRNA-FGF-21 (#2 and 3), and 2 μg TIF1γ. Each protein expression level was normalized to α-tubulin or β-actin. Statistical significance was determined using a one-way analysis of variance followed by Tukey’s multiple comparison test. Data are presented as mean ± SEM. *p < 0.05.

TIF1γ, Transcriptional intermediary factor 1 gamma; C2C12, Murine myoblast cells; BDNF, brain-derived neurotrophic factor; FGF-21, fibroblast growth factor 21; HK-2, human kidney 2 cells; EMT, epithelial-mesenchymal transition; CM, conditioned medium; siRNA, small interfering RNA; BMP-7, bone morphogenetic protein 7; α-SMA, alpha-smooth muscle actin; pSmad2/3, phosphorylated Smad2/3**.** ; SEM, standard error of the mean.
